# Hydrogen protects against liver injury during CO_2_ pneumoperitoneum in rats

**DOI:** 10.18632/oncotarget.23498

**Published:** 2017-12-15

**Authors:** Mingzi Chen, Lihong Jiang, Yue Li, Ge Bai, Jinghua Zhao, Ming Zhang, Jiantao Zhang

**Affiliations:** ^1^ Heilongjiang Key Laboratory for Laboratory Animals and Comparative Medicine, College of Veterinary Medicine, Northeast Agricultural University, Harbin 150030, China

**Keywords:** hydrogen, CO_2_ pneumoperitoneum, liver, ischemia-reperfusion

## Abstract

The aim of the current study was to identify the protective effect of hydrogen gas against liver injury during CO_2_ pneumoperitoneum. Rats were randomly divided into three groups: control group (C group), pneumoperitoneum group (P15 group) and hydrogen group (H_2_ group). Rats in the C group were subjected to anesthesia for 90 min. Rats in the P15 group received an abdominal insufflation of CO_2_ for 90 min at an intra-abdominal pressure of 15 mmHg. Rats in the H_2_ group received a hypodermic injection of hydrogen gas (0.2 mL/kg) and after 10 min they received an abdominal insufflation of CO_2_ for 90 min at an intra-abdominal pressure of 15 mmHg. Alanine aminotransferase (ALT) and aspartate aminotransferase (AST) were measured to evaluate liver function. Malondialdehyde (MDA), superoxide dismutase (SOD) and glutathione (GSH) content were measured to evaluate oxidative stress. Nuclear factor E2-related factor 2 (Nrf2) and Nrf2 downstream target genes, apoptosis-related genes and inflammatory cytokine mRNA and protein expression were detected. Liver injury was detected under the microscope. Our results revealed that liver function, antioxidants content, inflammation and liver injury were improved after hydrogen preconditioning in H_2_ group compared with P15 group. Overall, our results revealed that subcutaneous hydrogen injection could exert a protective effect against liver injury during CO_2_ pneumoperitoneum through reducing oxidative stress, cell apoptosis and inflammatory cytokines release.

## INTRODUCTION

Laparoscopic surgery is a procedure largely preferred than traditional surgery because of its several advantages including slight hemorrhage, less body injury due to small incisions and shorter recovery time [1]. Nevertheless, this procedure is not free from adverse effects and many of them are still under investigation.

To perform laparoscopic surgery the insufflation of a gas is required to induce pneumoperitoneum that provide enough working space inside the abdomen and a better visibility of the internal organs and tissues. At present, carbon dioxide (CO_2_) is the most commonly used gas to induce pneumoperitoneum [2]. The usual pressure value (11–14mmHg) of pneumoperitoneum is higher than the normal portal blood pressure of 7–10 mmHg [3]. High pressure can cause reduction in liver blood flow which results in oxidative stress and tissue injury [4, 5]. The deflation restores the normal liver blood circulation, but it results in ischemia-reperfusion injury [6].

Liver ischemia induces oxidative stress and reactive oxygen species (ROS) are generated during reperfusion, resulting in a serious liver injury [7]. In addition to oxidative stress, the mechanisms of ischemia reperfusion injury include cell apoptosis and cytokine/chemokine release [8–10]. Although ischemia reperfusion injury is due to multiple factors, ROS play a key role in inducing this injury [11–13]. Several studies show that hydrogen reduces ROS by exerting an antioxidant action, thus protecting against ischemia-reperfusion injury [14–16]. As a therapeutic medical gas, hydrogen can mitigate many diseases including parkinson's disease, acute pancreatitis, obstructive jaundice and sepsis [17–20]. Moreover, hydrogen protects against tissue damage induced by ischemia-reperfusion by reducing oxidative stress, cell apoptosis and inflammatory cytokines release [21–24]. However, no studies evaluating the protective effects of hydrogen to reduce liver injury during pneumoperitoneum are available. Therefore, the present study was performed to investigate the protective effects of hydrogen on liver during pneumoperitoneum.

## RESULTS

### Hypodermic injection of hydrogen gas protected against liver injury induced by pneumoperitoneum

**Figure 1:**
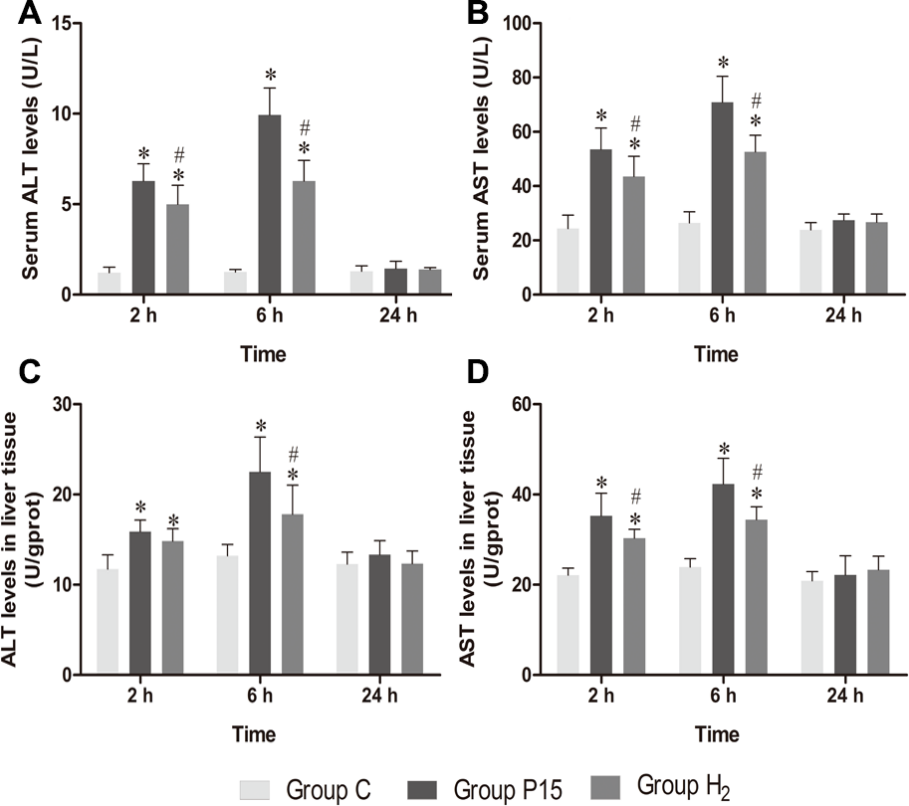
Hypodermic injection of hydrogen gas protected against liver injury induced by pneumoperitoneum (**A**, **B**) The ALT and AST levels in serum. (**C**, **D**) The AST and ALT levels in liver tissue. Data are presented as mean ± SD. ^*^p < 0.05 *vs* C group. ^#^*p* < 0.05 *vs* P15 group.

Serum AST and ALT levels increased in P15 and H_2_ group compared with C group at 2 h and 6 h. However, their levels were significantly reduced in H_2_ group compared with P15 group (*P* < 0.05, Figure 1A and 1B). AST and ALT levels in liver tissue showed the same trend (*P* < 0.05, Figure 1C and 1D).

### Hypodermic injection of hydrogen gas protected against liver oxidative stress injury induced by pneumoperitoneum

**Figure 2:**
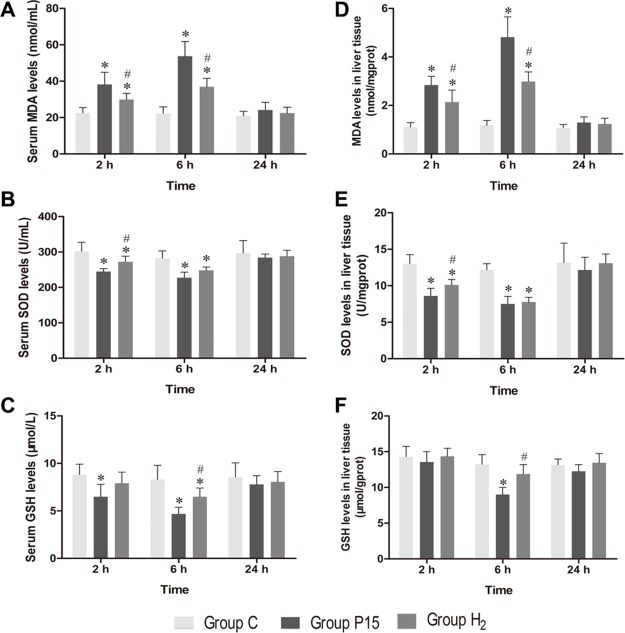
Hypodermic injection of hydrogen gas protected against liver oxidative stress injury induced by pneumoperitoneum (**A**, **B** and **C**) The MDA, SOD and GSH levels in serum. (**D**, **E** and **F**) The MDA, SOD and GSH levels in liver tissue. Data are presented as mean ± SD. ^*^p < 0.05 *vs* C group. ^#^*p* < 0.05 *vs* P15 group.

MDA, SOD and GSH concentrations in serum and liver tissue homogenate showed a similar trend among each other. MDA concentration significantly increased in P15 group compared with C group at 2 h and 6 h, while its concentration is significantly reduced in H_2_ group compared with P15 group (*P* < 0.05, Figure 2A and 2D). SOD concentration significantly decreased in P15 group compared with C group at 2 h and 6 h, while its concentration significantly increased in H_2_ group compared with P15 group (*P* < 0.05, Figure 2B and 2E) at 2 h. GSH concentration significantly decreased in P15 group compared with C group at 6 h, while its concentration significantly increased in H_2_ group compared with P15 group (*P* < 0.05, Figure 2C and 2F) at 6 h.

### Hypodermic injection of hydrogen gas protected against liver histology injury induced by pneumoperitoneum

**Figure 3:**
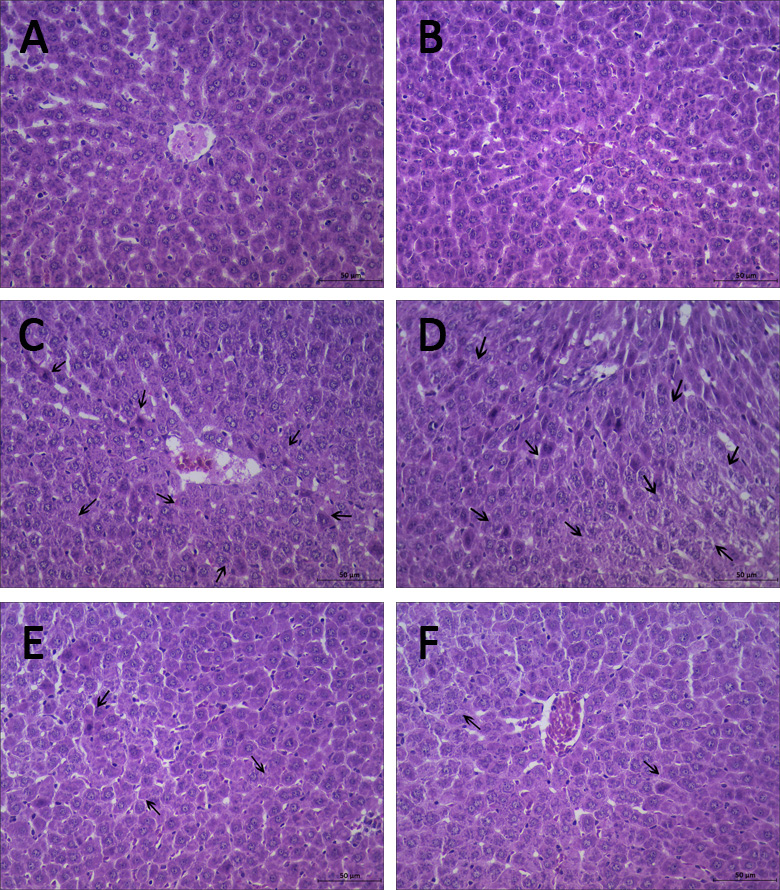
Hypodermic injection of hydrogen gas protected against liver histology injury induced by pneumoperitoneum Histopathology examination at 6 h. (**A**, **B**) C group. (**C**, **D**) P15 group. (**E**, **F**) H_2_ group.

Examination under the light microscope at 6 h revealed no significant pathological changes in C group (Figure 3A and 3B). Differences were observed in liver histology between P15 group and H_2_ group. Hepatocytes’ nuclei pyknosis, hepatocytes swelling and vacuoles degeneration were observed in P15 group (Figure 3C and 3D). In contrast, in H_2_ group, similar changes were found but in lesser degree (Figure 3E and 3F).

### Hypodermic injection of hydrogen gas activated Nrf2 signaling pathway

**Figure 4:**
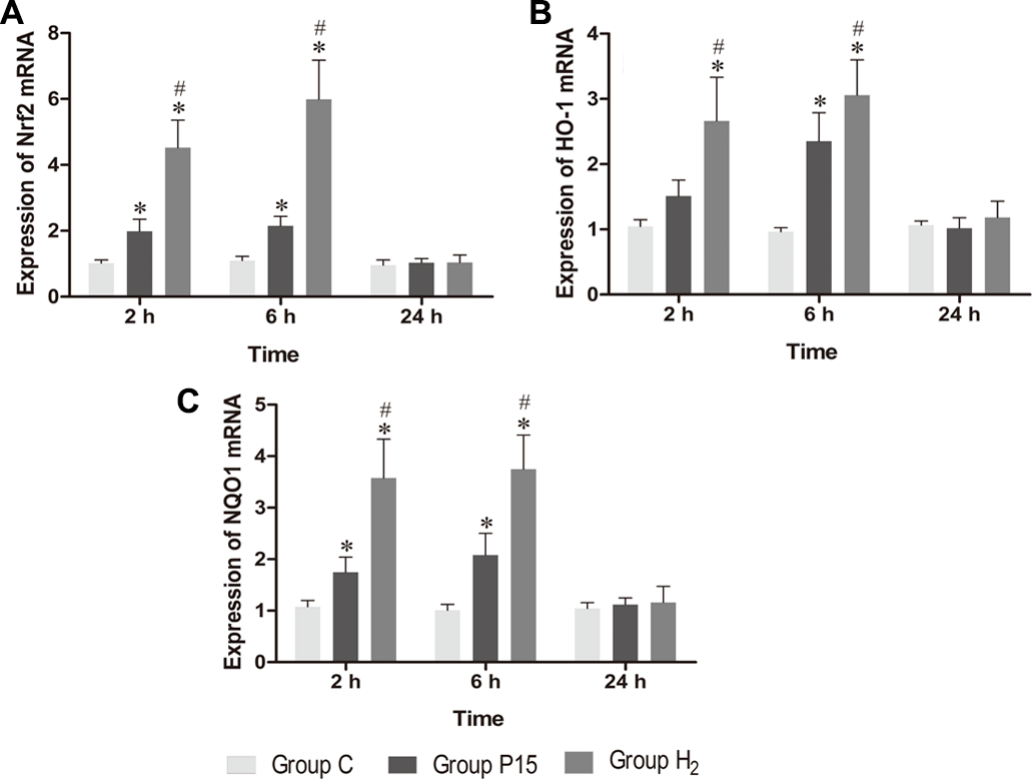
Hypodermic injection of hydrogen gas activated Nrf2 signaling pathway The Nrf2, HO-1 and NQO1 mRNA expression in liver. Data are presented as mean ± SD. ^*^p < 0.05 vs C group. ^#^*p* < 0.05 vs P15 group.

**Figure 5:**
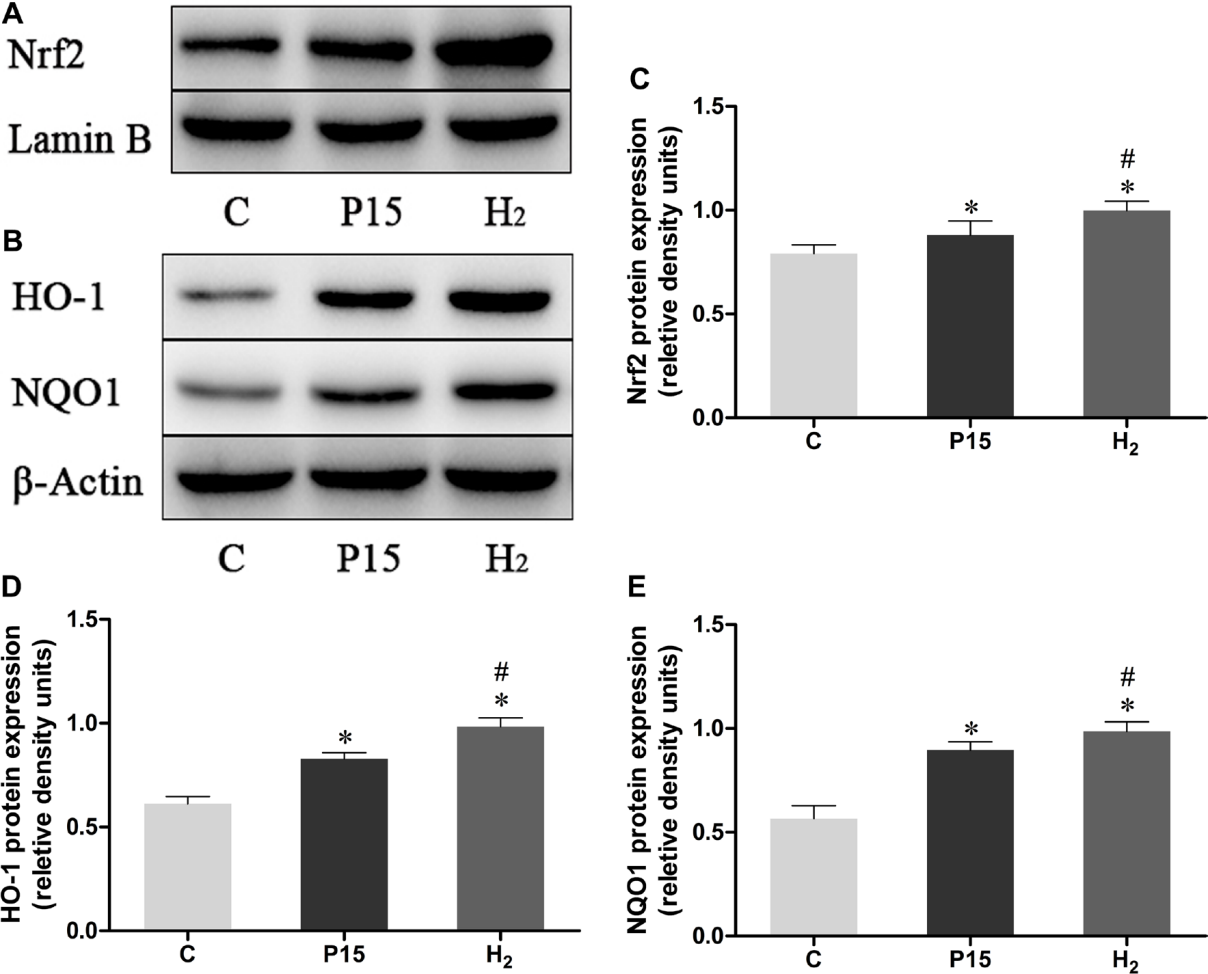
Hypodermic injection with hydrogen gas activated Nrf2 signaling pathway The Nrf2, HO-1 and NQO1 protein expression in liver at 6 h. Data are presented as mean ± SD. ^*^p < 0.05 vs C group. ^#^*p* < 0.05 vs P15 group.

Nrf2 mRNA expression was increased after pneumoperitoneum and hypodermic injection of hydrogen gas resulted in a further significant increase (*P* < 0.05, Figure 4A) at 2 h and 6 h. HO-1 and NQO1 mRNA expression were also examined using real-time PCR., Their expressions in H_2_ group remarkably increased compared with P15 group (*P* < 0.05, Figure 4B and 4C). Real-time PCR results were confirmed by western blot at 6 h (Figure 5).

### Hypodermic injection of hydrogen gas protected against liver injury induced by pneumoperitoneum by reducing apoptosis

**Figure 6:**
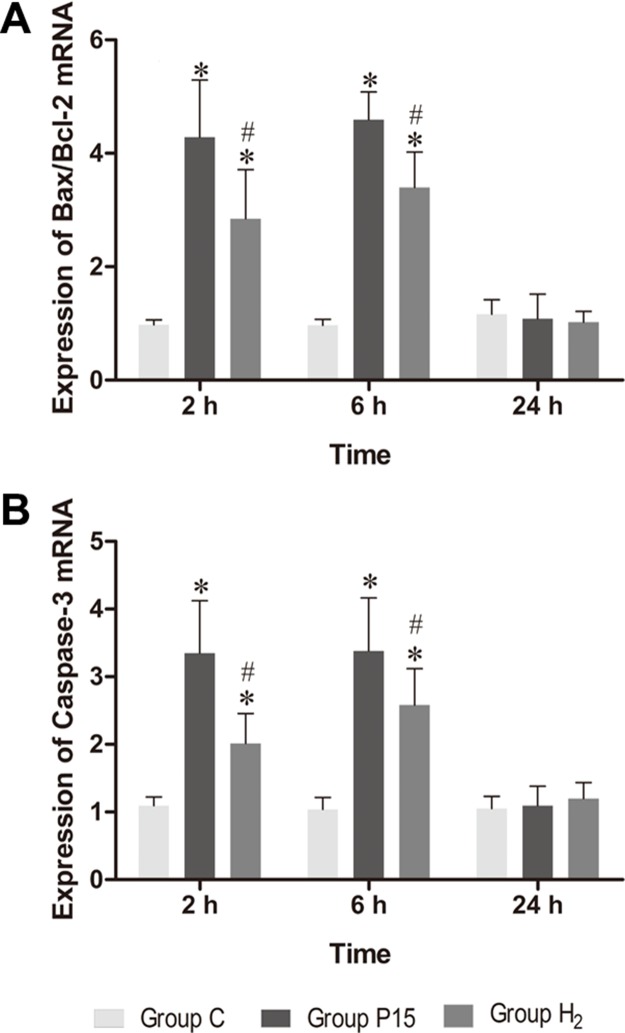
Hypodermic injection of hydrogen gas protected against liver injury induced by pneumoperitoneum by reducing apoptosis The Bax/Bcl-2 and Caspase-3 mRNA expression in liver. Data are presented as mean ± SD. ^*^p < 0.05 vs C group. ^#^*p* < 0.05 vs P15 group.

**Figure 7:**
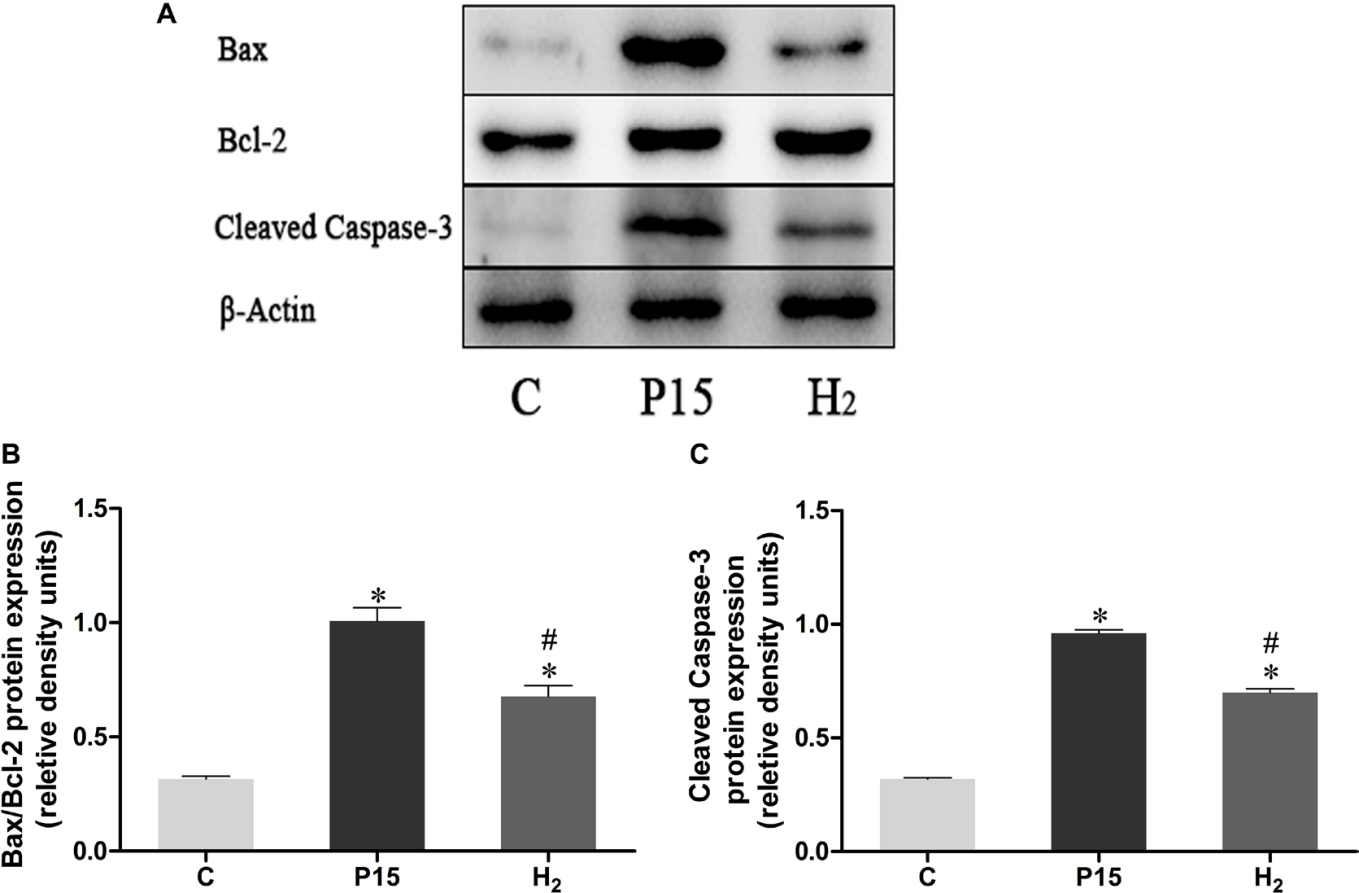
Hypodermic injection of hydrogen gas protected against liver injury induced by pneumoperitoneum by reducing apoptosis The Bax/Bcl-2 and Cleaved Caspase-3 protein expression in liver at 6 h. Data are presented as mean ± SD. ^*^p < 0.05 vs C group. ^#^*p* < 0.05 vs P15 group.

Bax/Bcl-2 and Caspase-3 mRNA expression was increased after pneumoperitoneum, while hypodermic injection of hydrogen gas resulted in a significant decrease (*P* < 0.05, Figure 6) at 2 h and 6 h. Real-time PCR results were confirmed by western blot at 6 h (Figure 7).

### Hypodermic injection of hydrogen gas protected against liver injury induced by pneumoperitoneum by reducing inflammatory cytokine production

**Figure 8:**
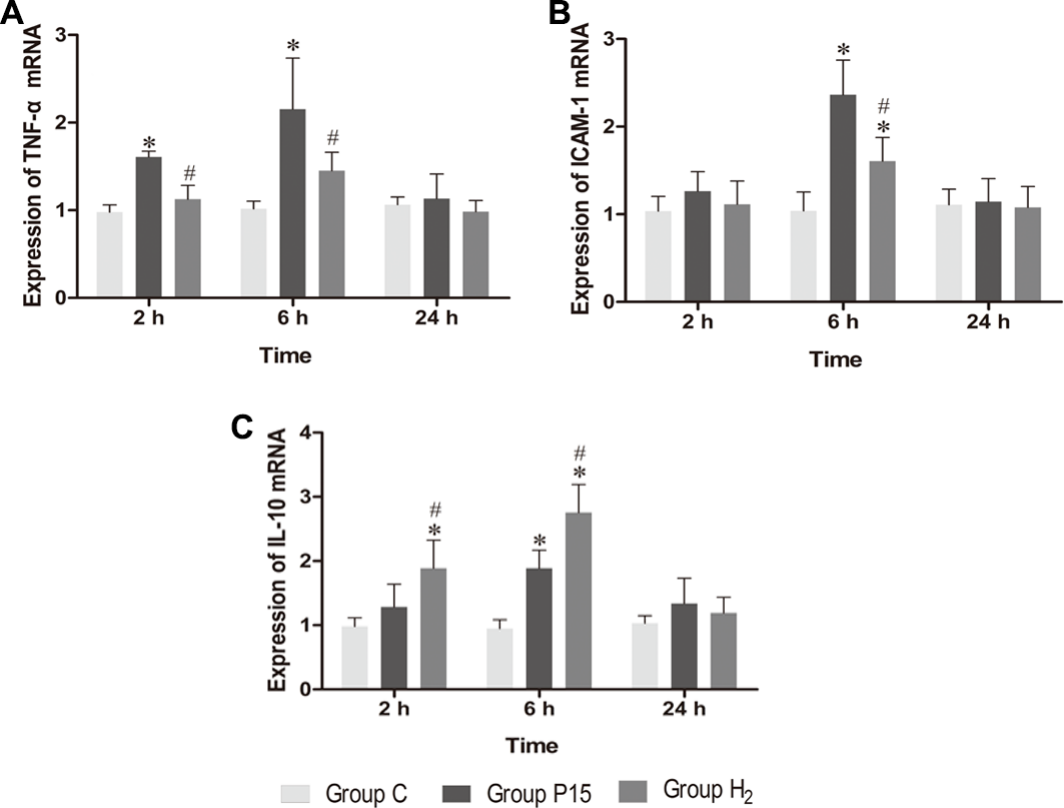
Hypodermic injection of hydrogen gas protected against liver injury induced by pneumoperitoneum by reducing inflammatory cytokine production The TNF-α, ICAM-1 and IL-10 mRNA expression in liver. Data are presented as mean ± SD. ^*^p < 0.05 vs C group. ^#^*p* < 0.05 vs P15 group.

**Figure 9:**
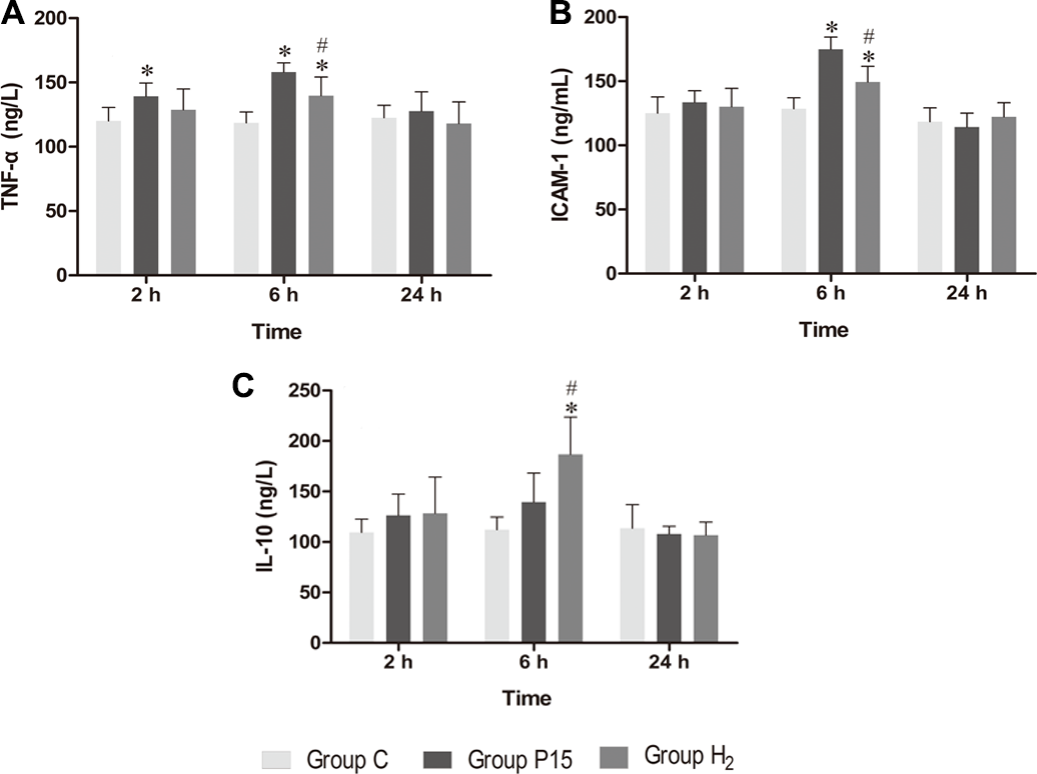
Hypodermic injection of hydrogen gas protected against liver injury induced by pneumoperitoneum by reducing inflammatory cytokine production The TNF-α, ICAM-1 and IL-10 protein expression in liver by ELISA. Data are presented as mean ± SD. ^*^p < 0.05 vs C group. ^#^*p* < 0.05 vs P15 group.

Real-time PCR was used to measure TNF-α, ICAM-1 and IL-10 mRNA in the liver, while ELISA was used to evaluate protein expression in the liver tissue homogenate. TNF-α and ICAM-1 mRNA levels were significantly decreased in H_2_ group compared with P15 group (*P* < 0.05, Figure 8A and 8B) at 6 h. IL-10 mRNA expression was increased after pneumoperitoneum, and hypodermic injection of hydrogen gas resulted in a further significant increase (*P* < 0.05, Figure 8C) at 2 h and 6 h. Similarly, TNF-α and ICAM-1 proteins in liver tissue homogenate were significantly lower (*P* < 0.05, Figure 9A and 9B) while IL-10 protein was significantly higher (*P* < 0.05, Figure 9C) in H_2_ group compared with P15 group at 6 h.

## DISCUSSION

This study was performed to determine the protective effect of hydrogen subcutaneous injection on CO_2_ pneumoperitoneum inducing liver injury. Pneumoperitoneum is required in laparoscopic surgery and CO_2_ is the most commonly used gas during pneumoperitoneum. CO_2_ insufflation can result in a significant ischemia of splanchnic organs, followed by increased reperfusion injury after deflation [25]. Thus, CO_2_ pneumoperitoneum can cause negative effects on body health. Splanchnic organs such as liver, kidneys, spleen, intestine, ovaries and testicles are susceptible to pneumoperitoneum adverse effects [6, 26–30]. The liver receives blood supply from the hepatic artery and portal vein and it is one of the most susceptible organs affected by ischemia. It has been reported that 10 mm Hg pneumoperitoneum caused significant decrease in hepatic arterial, portal, and microvascular perfusion [31]. In addition, pneumoperitoneum increase from 10 to 15 mm Hg can decrease liver flow by 39%, causing liver ischemia injury, and deflation after surgery resulting in blood reperfusion results in liver ischemia reperfusion injury [32, 33]. Several studies showed that CO_2_ pneumoperitoneum affects liver enzyme function and causes tissue injury [34, 35]. Our results showed a similar result. Indeed our results showed that ALT and AST levels were increased in both P15 and H_2_ group, although their levels in H_2_ group were lower than in P15 group. The histopathological examination reflecting the degree of liver injury showed that injury in H_2_ group was less than P15 group. Therefore, hydrogen subcutaneous injection could reduce liver injury during CO_2_ pneumoperitoneum.

The use of H_2_ results in many advantages. More than 38 diseases can be relieved by the biological effects of H_2_ [36]. The methods of introducing H_2_ into the body can be different and include hydrogen gas inhalation, hydrogen water ingestion, and hydrogen-rich saline injection [36]. It has been demonstrated that the protective effect of hydrogen subcutaneous injection is the same as the protective effect obtained by hydrogen water intraperitoneal injection [37]. Furthermore, subcutaneous injection of hydrogen is more convenient, safer and rapidly absorbed compared with hydrogen gas inhalation and hydrogen-rich saline injection. In our study, the results of the biochemical analysis and the histopathological examination showed that subcutaneous injection of hydrogen reduced liver injury induced by CO_2_ pneumoperitoneum. This protective effect was associated with reduced oxidative stress, apoptosis and inflammatory cytokine production.

Ischemia reperfusion injury producing excess of ROS, results in the downregulation of some endogenous antioxidant enzymes including SOD [38]. SOD is a metal enzyme with a specific biological catalytic function. Superoxide radical can be catalyzed by SOD and transformed into molecular oxygen or hydrogen peroxide. MDA originates from the decomposition of oxidizes protein and lipid peroxyl radicals, thus its content reflects the degrees of lipid peroxidation [39]. MDA can be a cause of oxidative stress as well as a result. SOD and MDA levels usually estimated anti-oxidative ability of the tissue [40]. GSH is an important endogenous antioxidant [41]. GSH protects tissues from oxidative stress injury through ROS detoxification [42]. Our results showed that hydrogen subcutaneous injection reduced liver oxidative stress by decreasing MDA levels and increasing SOD and GSH levels, suggesting a protective effect of hydrogen against oxidative stress. Nrf2 is a transcription factor which acts against oxidative stress injury by mediating the levels of endogenous antioxidants. Keap1 normally binds to Nrf2 to suppress it and keep it into the cytoplasm. However, Keap-1-Nrf2 complex is dissociated after activation of Nrf2 due to oxidative damage, enabling Nrf2 to translocate into to the nucleus. Here, it binds to the antioxidant response element (ARE) and activates the transcription of antioxidant enzymes, including HO-1 and NQO1 [43–45]. Recent studies have shown that Nrf2 can protect liver against ischemia reperfusion injury [46]. Both HO-1 and NQO1 are key response genes, and their activation can protect against oxidative damage in cells [47]. HO-1 exerts cytoprotection by inhibiting inflammation and apoptosis [48]. It can also degrade heme to CO, iron, and biliverdin, exerting an indirect anti-oxidative effect [49]. NQO1 is a flavoprotein and its flavin co-factor plays a key role in the direct scavenging of superoxide [50]. Therefore, NQO1 may directly exert an antioxidant protection. Our results showed that Nrf2, HO-1 and NQO1 mRNA and protein levels were increased by pneumoperitoneum. After subcutaneous injection of hydrogen, both their mRNA and protein levels were significantly increased suggesting an improved liver protection against ischemia reperfusion injury during CO_2_ pneumoperitoneum through the increase of the antioxidants level.

Nrf2 also has an anti-inflammatory ability [51]. Inflammatory response can lead to liver injury during ischemia-reperfusion [52]. Inflammatory response is associated with the release of proinflammatory cytokines. TNF-α is a proinflammatory cytokine, and it is the main cytokine acting in the acute-phase response [53]. ICAM-1 is a cell adhesion molecules that can be induced by TNF-α, while IL-10 is an anti-inflammatory cytokine that represses proinflammatory response, thus reducing tissue damage caused by inflammation [54, 55]. Previous studies suggest that elevated intra-abdominal pressure can enhance proinflammatory cytokine expression [56]. Indeed, Kupffer cells are activated at the initial phase of reperfusion and they consequently release both proinflammatory cytokines such as TNF-α and anti-inflammatory cytokines such as IL-10 [57, 58]. Furthermore, ischemia-reperfusion could increase ICAM-1 expression in liver endothelial cells [59]. In our study, IL-10, TNF-α and ICAM-1 expression was increased after pneumoperitoneum. However, hydrogen subcutaneous injection enhanced IL-10 mRNA and protein expression and reduced TNF-α and ICAM-1 mRNA and protein expression, indicating that hydrogen improved inflammation caused by CO_2_ pneumoperitoneum.

Apoptosis can be stimulated during liver ischemia-reperfusion injury [60]. Indeed, liver ischemia-reperfusion can increase the expression of Bax and cleaved caspase-3 [61]. Also a high CO_2_ pneumoperitoneum pressures causes an increased apoptosis [62]. Bax and Bcl-2 are important genes in cell apoptosis progress. Bax is a pro-apoptotic protein, while Bcl-2 is an anti-apoptotic protein. Bax can induce caspases activation among other various apoptotic molecules [63]. Cleaved caspase-3 plays an important role in the caspase cascade characterizing the apoptotic pathway. Activated caspase-3 leads to DNA fragmentation and apoptosis [64]. In our study, pneumoperitoneum did not increase Bcl-2 expression, but it increased Bax and cleaved caspase-3 expression, suggesting that pneumoperitoneum induced hepatocyte apoptosis. After hydrogen subcutaneous injection, Bax and cleaved caspase-3 expression was reduced, indicating a decreased hepatocyte apoptosis during CO_2_ pneumoperitoneum.

Some methods are available to reduce pneumoperitoneum-induced injury such as ischemic-preconditioning, pneumoperitoneum preconditioning, low intra-abdominal pressure, stepwise rising CO_2_ insufflation, melatonin, theophylline, erythropoietin and dexmedetomidine [65–71]. Some agents are also available to reduce ischemia-reperfusion injury such as epigallocatechin-3-gallate, gastrodin, eupatilin and oxymatrine [38, 72–74]. If H_2_ can be used in clinical practice it would be convenient because it is cheaper compared with those methods, although its inhalation may be dangerous because of its inflammable and explosive characteristics. Subcutaneous injection of hydrogen was safer and simpler than inhalation. Moreover, H_2_ does not leave residues compared with other drugs. As a potential antioxidant, H_2_ has many advantages such as rapid diffusion, no direct elimination of functionally important ROS, no toxicity even at higher concentration [36]. Therefore our study highlighted that subcutaneous injection of hydrogen could exert a protective effect on liver injury during CO_2_ pneumoperitoneum through reducing oxidative stress, cell apoptosis and inflammatory cytokines release. Hence, subcutaneous injection of hydrogen might be a promising approach against pneumoperitoneum-induced livers injury.

## MATERIALS AND METHODS

### Animals

Forty-five male Wistar rats, weighing 250–300 g, were housed under an alternating 12-h light/12-h dark cycle and free access to standard rat food and water; the environmental conditions were constant. Food was withheld 12 h prior to anesthesia, but free access to water was allowed. All experimental procedures were performed according to the guidelines approved by the Northeast Agricultural University, Harbin, China.

### Surgical technique

Forty-five rats were randomly divided into three groups (*n* = 15). Rats were anaesthetized at the beginning of the experiment using isoflurane inhalation in a 1.5% mixture of oxygen, delivered using an animal anesthetic respiratory system (Surgivet Co., Ltd, USA). After anesthesia was performed, animals were placed in a supine position, and the abdominal skin was disinfected. Hydrogen gas was provided by a hydrogen generator (Saikesaisi hydrogen energy Co., Ltd, Shandong, China). Pneumoperitoneum was generated in all animals, except the control group, by inserting a 22G I.V. cannula needle. CO_2_ was insufflated using an automatic device (*Olympus* Co., Ltd, JPN) at a low rate until the intra-abdominal pressure reached 15 mmHg. Rats in the control group (C group) were subjected only to anesthesia for 90 min. Rats in the pneumoperitoneum group (P15 group) received an abdominal insufflation of CO_2_ for 90 min at an intra-abdominal pressure of 15 mmHg. Rats in the H_2_ group received a hypodermic injection of hydrogen gas (0.2 mL/kg) and after 10 min they received an abdominal insufflation of CO_2_ for 90 min at an intra-abdominal pressure of 15 mm Hg.

### Blood and tissue samples collection

Rats were sacrificed by decapitation at 2 h, 6 h and 24 h after deflation. Blood samples were collected and centrifuged at 3500 rpm for 10 min and serums were stored at -20°C until analysis. A part of liver tissue was placed in 10% formalin for histological studies. Another part was homogenized in ice-cold saline and centrifuged at 2500 rpm for 10 min and supernatants were stored at -20°C until analysis. A third part was frozen immediately in liquid nitrogen and stored at −80°C until analysis.

### Biochemical analysis

MDA, SOD, GSH, AST and ALT in serums and liver tissue homogenates were measured using the respective detection kits (Nanjing Jiancheng Bioengineering Institute, Nanjing, China) according to the manufacturer's instructions.

### ELISA

TNF-α, ICAM-1 and IL-10 in the liver tissue homogenate were determined using the respective ELISA kits (Nanjing Jiancheng Bioengineering Institute, Nanjing, China) as described in the instructions. Absorbance was measured at 450 nm according to a standard curve and cytokines were expressed as ng/L or ng/mL.

### Real-time RT-PCR

Total RNA was extracted from the liver using Trizol reagent (Invitrogen, Carlsbad, USA) as described in the instructions. cDNA synthesis was performed in 1 μg of total RNA using PrimeScript™ RT reagent Kit with gDNA Eraser (Takara, Dailian, China) as described in the instructions. Quantitative real-time PCR was performed in LightCycler 2.0 (Roche Applied Science, Penzberg, Germany) using the SYBR® Premix Ex Taq™ II (Takara, Dailian, China) as described in the instructions. Primers (Table 1) were synthesized by Takara (Dailian, China). Reaction parameter was: 1 cycle of 95°C for 30 s, 45 cycles of 95°C for 5 s, primer-specific annealing temperature for 20 s, and extension at 72°C for 20 s. The relative mRNA expression was analyzed using the 2^−ΔΔCt^ method.

**Table 1:** Primer sequences

Gene	Primer Sequence (5′-3′)
Nrf2	GAGACGGCCATGACTGAT (forward)
	GTGAGGGGATCGATGAGTAA (reverse)
HO-1	ATCGTGCTCGCATGAAC (forward)
	CAGCTCCTCAAACAGCTCAA (reverse)
NQO1	CAGCGGCTCCATGTACT (forward)
	GACCTGGAAGCCACAGAAG (reverse)
Bcl-2	GGGATGCCTTTGTGGAACTA (forward)
	CTCACTTGTGGCCCAGGTAT (reverse)
Bax	TGTTTGCTGATGGCAACTTC (forward)
	GATCAGCTCGGGCACTTTAG (reverse)
Caspase-3	ACAGAGCTGGACTGCGGTAT (forward)
	TGCGGTAGAGTAAGCATACAGG (reverse)
TNF-α	GGCCACCACGCTCTTCTGTC (forward)
	GGGCTACGGGCTTGTCACTC (reverse)
ICAM-1	AAACGGGAGATGAATGGT (forward)
	TCTTCCTCTGGCGGTAAT (reverse)
IL-10	TGGACAACATACTGCTGACAG (forward)
	GGTAAAACTTGATCATTTCTGACAAG (reverse)
Actb	GTCGTACCACTGGCATTGTG (forward)
	CTCTCAGCTGTGGTGGTGAA (reverse)

### Western blot

Frozen liver tissues were homogenized in ice-cold RIPA Lysis Buffer (Beyotime Biotechnology, Nanjing, China) or Nuclear and Cytoplasmic Protein Extraction Kit (Beyotime Biotechnology, Nanjing, China) containing PMSF (Beyotime Biotechnology, Nanjing, China) as described in the instructions. The homogenization was centrifuged at 12,000 × g for 20 min at 4°C. The supernatant was collected and protein concentration was determined using the Enhanced BCA Protein Assay Kit (Beyotime Biotechnology, Nanjing, China) as described in the instructions. Protein samples (30 μg) were denatured for 5 min at 100°C in 5× SDS-PAGE Sample Loading Buffer (Beyotime Biotechnology, Nanjing, China). Electrophoresis was performed using SDS-PAGE Gel Quick Preparation Kit (Beyotime Biotechnology, Nanjing, China), 10% acrylamide gels for β-actin, NRF2, Lamin B, and 12% acrylamide gels for HO-1, NQO1, Bcl-2, Bax, and 15% acrylamide gels for Cleaved Caspase-3. Protein were transferred to nitrocellulose membranes using the VE 186 Trans-Blot apparatus (Tanon, Shanghai, China). Next, they were blocked for 2 h with 5 % skim milk at room temperature. Follow by the membranes were incubated with primary antibodies overnight at 4°C. The primary antibodies were used at the following dilutions: anti-β-actin diluted 1:5000 (ABclonal, Wuhan, China), anti-Nrf2 diluted 1:1000 (ABclonal, Wuhan, China), anti-Nrf2 diluted 1:1000 (ABclonal, Wuhan, China), anti-HO-1 and NQO1 diluted 1:25000 (Abcam, MA, USA), anti-Lamin B, Bcl-2, Bax and Cleaved Caspase-3 diluted 1:1000 (Wanlei, Shenyang, China). Membranes were incubated with secondary antibody (dilution 1:5000, Zhongshan Goldenbridge Biotech, Beijing, China) for 2 hour at room temperature. The signals were visualized using the ECL kits (Beyotime Biotechnology, Nanjing, China). The blots were scanned using a Tanon 5200 Imaging System (Tanon Science & Technology Co., Shanghai, China).

### Histopathology examination

Livers in 10% formalin were embedded in paraffin, cut into sections and stained with hematoxylin and eosin. All specimens were examined under light microscope.

### Statistical analysis

Data are presented as mean ± standard deviation (SD). Statistical analysis was performed using SPSS version 22.0. A value of *p* < 0.05 was considered statistically significant.

## Abbreviations

ALT: Alanine aminotransferase
AST: aspartate aminotransferase
MDA: malondialdehyde
SOD: superoxide dismutase
GSH: glutathione
Nrf2: nuclear factor E2-related factor 2
HO-1: Heme oxygenase-1
NQO1: NAD(P)H quinone dehydrogenase 1
BCL-2: B cell lymphoma/lewkmia-2
Bax: Bcl-2 assaciated x protein
Caspase-3: cysteinyl aspartate specific proteinase 3
TNF-α: tumor necrosis factor α
ICAM-1: intercellular cell adhesion molecule-1
IL-10: interlenkin-10.
